# Improved Health among People Living with HIV/AIDS Who Received Packages of Proven Preventive Health Interventions, Amhara, Ethiopia

**DOI:** 10.1371/journal.pone.0107662

**Published:** 2014-09-18

**Authors:** Ciara E. O’Reilly, Ethel V. Taylor, Tracy Ayers, Ribka Fantu, Sisay Alemayehu Abayneh, Barbara Marston, Yordanos B. Molla, Tegene Sewnet, Fitsum Abebe, Robert M. Hoekstra, Robert Quick

**Affiliations:** 1 Division of Foodborne, Waterborne, and Environmental Diseases, Centers for Disease Control and Prevention, Atlanta, Georgia, United States of America; 2 Epidemic Intelligence Service, Centers for Disease Control and Prevention, Atlanta, Georgia, United States of America; 3 Centers for Disease Control and Prevention, Addis Ababa, Ethiopia; 4 Division of Global HIV/AIDS, Centers for Disease Control and Prevention, Atlanta, Georgia, United States of America; 5 Michael Dejene Public Health Consultancy Services, Addis Ababa, Ethiopia; China Medical University, China

## Abstract

In 2009, basic care packages (BCP) containing health products were distributed to HIV-infected persons in Ethiopia who were clients of antiretroviral therapy clinics. To measure health impact, we enrolled clients from an intervention hospital and comparison hospital, and then conducted a baseline survey, and 7 bi-weekly home visits. We enrolled 405 intervention group clients and 344 comparison clients. Intervention clients were more likely than comparison clients to have detectable chlorine in stored water (40% vs. 1%, p<0.001), soap (51% vs. 36%, p<0.001), and a BCP water container (65% vs. 0%, p<0.001) at every home visit. Intervention clients were less likely than comparison clients to report illness (44% vs. 67%, p<0.001) or health facility visits for illness (74% vs. 95%, p<0.001), and had lower median illness scores (1.0 vs. 3.0, p<0.05). Participation in the BCP program appeared to improve reported health outcomes.

## Introduction

Diarrhea and other opportunistic infections are important causes of morbidity and mortality among people living with HIV/AIDS (PLHIV) [1]–[5]. Inexpensive interventions to prevent opportunistic infections in resource-poor settings can improve clinical outcomes in PLHIV [5]–[11] and may help reduce viral loads and maintain CD4+ cell counts (CD4 counts) [12]–[15]. Consequently, the US President’s Emergency Plan for AIDS Relief (PEPFAR) now authorizes use of program funds to procure and distribute proven health interventions (e.g., insecticide treated bednets, condoms, household water treatment products, soap) as part of HIV care and support programs [16].

Among the interventions shown to reduce the risk of diarrhea in PLHIV is household water treatment using a locally produced sodium hypochlorite solution and safe water storage using a narrow-mouth container with tap [5], [7]. In Ethiopia, two water treatment products for home chlorination are available: sodium hypochlorite solution with the brand name Wuha Agar, which is marketed at a price of USD 0.24 for a bottle that treats 1,000 liters of water, and a flocculent-disinfectant product with the brand name PuR that clarifies and disinfects water [17], and is available in Ethiopia at a price of USD 0.06 for a sachet that treats 10 liters.

In June 2009, with PEPFAR funding provided though the United States Agency for International Development (USAID) program, the Ministry of Health of Ethiopia and Population Services International (PSI) began a program to distribute basic preventive care packages (BCPs) [18], [19] to HIV-infected persons receiving antiretroviral therapy (ART). The BCPs were distributed through a phased roll-out to clinics in five regions of Ethiopia: Tigray, Amhara, Oromia, Southern Nations, Nationalities and Peoples (SNNP), and Addis Ababa. Each BCP included: 6 bottles of Wuha Agar (enough to treat 20 liters of water/day for 6 months), 24 sachets of PuR (enough to treat 10 liters of water/day for 24 days), a 20 L jerrican with a lid and tap for safe water storage, 100 condoms, 4 bars of soap, 4 doses of albendazole (enough for one dose per quarter), and a low literacy illustrated information booklet in Amharic promoting appropriate water treatment, sanitation and hygiene practices. Cotrimoxazole prophylaxis was provided free to clients at ART clinics when indicated, as per Ethiopian Federal Ministry of Health guidelines [20]. Distribution of the BCP to clients was accompanied by demonstration of product use by trained hospital staff. All products contained in the BCP, except for the water storage container, were available on the market in Ethiopia before the BCP program began.

During the last quarter of 2009, 75,000 BCPs were distributed, with another 125,000 BCPs distributed in 2010 and 2011. The expense and effort to implement this program warrants an evaluation of impact. From September 2009 through January 2010, we assessed the use, acceptability, and health impact of the BCP on ART clients in Amhara, the region with the highest proportion of patients receiving ART in Ethiopia as a consequence of its large population size, and the high adult HIV prevalence rates (approximately 4.5%: 3.2% in rural and 13.5% in urban settings) [21], [22].

## Materials and Methods

### Study Population and Sample Size Calculations

Using previously published methods [5], [7] we conducted a longitudinal evaluation of the BCP program among PLHIV (hereafter referred to as clients) enrolled in ART programs. Because of resource constraints, this evaluation was limited to a convenience sample of two health facilities in Amhara Region, Ethiopia. We selected the health facilities based on similar numbers of clients currently receiving ART, demographic characteristics, estimated risk for diarrheal disease, and, for logistical reasons, ease of access by road. The Amhara Regional Health Bureau designated Gonder University Hospital, a large teaching hospital under the administration of the Department of Education that provides treatment for 3,175 ART clients, as the intervention site and Debre Markos Zonal Hospital, a large regional hospital under the auspices of the Ethiopian Ministry of Health that provides treatment for 2,056 ART clients, as the comparison site. The ART programs in both of these facilities were supported by International Training & Education Center for Health (ITEC, Seattle, Washington). Even though Gonder Hospital is an academic hospital, the HIV clinics at both hospitals are run by ITEC as the PEPFAR contracted partner organization. The two sites were similar in terms of clinical oversight, located in a town, access to the health facility (all participants lived within 10 km of the health facility), geography, climate, and ethnic group representation (>95% of participants at both sites were of Amhara ethnicity). Both communities are at altitudes >2,000 m (i.e. not at high risk of malaria transmission [Gonder 2,133 m; Debre Markos 2,446 m]) and have similar climates.

A sample size of approximately 400 persons each in the intervention and comparison groups was estimated to detect a 10% reduction in illnesses in the intervention group relative to the comparison group, with 80% power, 95% confidence, and a 25% drop out rate. We limited participation to one client per household.

Adult clients (≥18 years old) enrolled in the HIV care and treatment program and receiving ART treatment who lived within a 10 km radius of the selected health facilities were eligible for inclusion. Because records lacked sufficient information to locate patients and daily patient load was relatively low, random selection of participants was not feasible. Clients who consented to participate were consecutively enrolled during clinic visits until the target sample size was reached or the eligible client population was exhausted.

### Baseline Survey

Field workers administered a baseline questionnaire in Amharic from September 24–October 23, 2009 to participants at both health facilities to gather information about demographic and socioeconomic characteristics; water, sanitation and hygiene knowledge and practices; and utilization rates of BCP components. Clinic nurses at both health facilities also abstracted medical information from clients’ hospital charts including the most recently recorded CD4 count, functional status, prescription of cotrimoxazole prophylaxis, and duration of ART. Intervention group clients received the BCP after completion of the baseline questionnaire. Fieldworkers accompanied clients to their homes after the baseline interview to make observations on water treatment, storage, and hygiene and sanitation facilities, and to test stored drinking water for free residual chlorine using the N, N-diethyl-p-phenylenediamine (DPD) colorimetric method (LaMotte Co., Baltimore, MD) as an objective measure of water treatment.

### Biweekly Home Visits

Following the baseline survey, field teams began biweekly home visits to study participants from both groups on October 8, 2010 to ask about water, sanitation and hygiene practices; ongoing use of BCP components and cotrimoxazole prophylaxis; illnesses in the 48 hours preceding the visit; health facility visits or hospitalizations in the previous 14 days; and perceived BCP-associated stigma among clients in the intervention group. Fieldworkers tested stored drinking water for free residual chlorine and made observations of BCP components and cotrimoxazole tablets in the home. Deaths among study participants were also recorded. Biweekly home visits continued for seven rounds. Comparison group clients received the BCP at the end of the study, in January 2010.

### Definitions and Statistical Analysis

We defined diarrhea as ≥3 loose or watery stools in a 24 hour period; respiratory infection as reported fever and ≥2 of the following signs and symptoms: cough, difficulty breathing, or respiratory rate >20 breaths per minute; and febrile illness as reported subjective fever without concurrent signs and symptoms of diarrhea or respiratory illness. Additionally, we calculated a total illness score for each client that summed all illnesses reported for the previous 48 hours at each home visit and all reported health facility visits or hospitalizations for any illness. Illnesses spanning multiple home visits were counted each time they were reported.

Data were double-entered in Ethiopia into EPI Info (CDC, Atlanta, GA, USA). Data analysis was performed using SAS version 9.2 (SAS Institute, Cary, NC, USA). Product use and illness data between groups were compared using Chi-Square tests for categorical variables. We performed categorical data analysis on continuous variables based on natural breakpoints within the data and compared differences between the two groups using Chi-Square tests. The total illness score was weighted to account for severity of illness, with self-reported health facility visits for illness or hospitalizations contributing double the value of self-reported acute illnesses in the previous 48 hours for which clients did not seek care. Additional weighting factors for health facility visits and hospitalizations were also evaluated, with no impact on outcomes, therefore the most conservative weighting factors were chosen. Health facility visits for HIV follow-up care (e.g., ART refills, blood testing for CD4 counts) were not included in the total illness score. Because total illness scores were not normally distributed, we used the Wilcoxon rank-sum test to test for statistical significance. Estimation of correlation structures within individuals over time and across all measured variables was not appropriate in this study due to the lack of stability in such estimates that can arise from the relatively small sample size, weak correlation, and highly skewed distributions. In collapsing the data over time, we lost power, but gained simplicity and robustness. We choose to present a common analysis for all variables that facilitates comparisons, although for some variables the data were sufficient to incorporate correlation structures in the analysis; the scientific findings were not altered by this approach.

### Ethical Review

The study protocol was approved by an Institutional Review Board of the Centers for Disease Control and Prevention, Atlanta, GA, USA (Protocol Number 5543), and the Ethiopian Public Health Association (Protocol Number IERC 0030). Written informed consent was obtained at the beginning of the study from all participants.

## Results

We enrolled 405 clients in the intervention group and 344 clients in the comparison group. Overall, 26 (0.3%) clients refused to participate. We excluded from analysis 29 (7%) intervention group clients and 36 (10%) comparison group clients who did not complete all rounds of biweekly surveillance, including 8 who died (3 in the intervention and 5 in the comparison group). The reported cause of death as per the medical chart in the intervention group clients was suicide (1), pneumonia (1), unknown cause (1), and in the control group was car accident (1), chronic liver disease and pulmonary tuberculosis (1), diarrhea/vomiting (2), pulmonary tuberculosis and pneumonia (1).

### Baseline Survey

The mean age of study participants was 36 years (range 19–69 years); 75% were female (Table 1). Over a third of participants had no formal schooling, and more than 65% earned no income. Nearly 70% of clients lived in a rented home and over 80% lived in households with 5 or fewer members. There were no significant differences in demographic characteristics between intervention and comparison group clients.

**Table 1:** Baseline demographic characteristics of study participants in intervention and comparison groups, basic care package evaluation, Gonder and Debre Markos, Ethiopia, October 2009.

Characteristic		InterventionGroup (n = 376)	ComparisonGroup (n = 308)	p-value	Overall(n = 684)
Mean age ± SD^a^		35.6±9.5	37.0±10.0	0.05	36.2±9.7
Age group, n (%)^b^	18–25 years	49 (12.1)	43 (12.5)	0.7	92 (12.3)
	26–35 years	193 (47.7)	145 (42.2)	0.3	338 (45.1)
	36–45 years	101 (24.9)	88 (25.6)	0.6	189 (25.2)
	>45 years	62 (15.3)	68 (19.8)	0.06	130 (17.4)
Female, n (%)^b^		278 (73.9)	234 (75.9)	0.6	512 (74.9)
Ethnicity, n (%)	Amhara^b^	360 (95.7)	302 (98.1)	0.1	662 (96.8)
	Tigray^c^	10 (1.5)	2 (0.7)	0.08	12 (1.8)
	Oromo^c^	2 (0.5)	3 (0.8)	0.7	5 (0.7)
No formal schooling, n (%)^b^		121 (32.2)	109 (35.4)	0.4	230 (33.6)
Do not earn income, n (%)^b^		254 (67.6)	200 (64.9)	0.5	454 (66.4)
Living in rented house, n (%)^b^		260 (69.2)	212 (68.8)	0.9	472 (69.0)
Number of persons living in householdin past six months, n (%)^b^	1 to 5	298 (79.3)	269 (87.3)	<0.05	567 (82.9)
	>5	78 (20.7)	39 (12.7)	<0.05	117 (17.1)

Comparison between intervention and comparison groups using Kruskal-Wallis test^a^, Chi-Square test^b^, or Fisher exact test^c^.

At baseline, more than 95% of clients in both groups were assessed by ART clinical staff as able to work (Table 2). At the time of enrollment, 81% of clients in the intervention group and 77% in the comparison group had been on ART for >12 months. Based on abstracted medical records, >99% of clients in both groups had good adherence to ART (<5% of doses missed). Most recently recorded median CD4 counts were similar at baseline for both intervention and comparison group clients (284 and 274 cells/µL, respectively). The range of CD4 counts in the intervention and control groups respectively were 26% vs. 33% <200 cells/µL, 38% vs. 35% 200–<350 cells/µL, 20% vs. 19% 350–>500 cells/µL, and 17% vs. 12% ≥500 cells/µL. A higher percentage of clients in the comparison group than in the intervention group were receiving cotrimoxazole prophylaxis (79% vs. 68%, p<0.05) at baseline.

**Table 2:** Clinical characteristics and treatment of ART clients at baseline, by study group, basic care package evaluation, Gonder and Debre Markos, Ethiopia, October 2009.

Characteristic		InterventionGroup (n = 376)	ComparisonGroup (n = 308)
**Functional status, n (%)**			
	Ambulatory	11 (3.0)	13 (4.2)
	Working	361(97.0)	295 (95.8)
**CD4 count in cells/µL, median (range)**		284 (3–996)	274 (31–1088)
**Time on ART treatment in months, median (range)**		28.5 (0.1–67.3)	24.6 (0.2–73.2)
**>12 months on ART treatment, n (%)**		304 (81.1)	238 (77.3)
**Adherence to ART drugs** a **, n (%)**			
	Good	372 (99.2)	306 (99.4)
	Fair	3 (0.8)	1 (0.3)
	Poor	0	1 (0.3)
**Currently on cotrimoxazole** b **, n (%)**		256 (68.3)	243 (78.9)
**Adherence to cotrimoxazole** a **, n (%)**			
	Good	253 (98.8)	240 (98.8)
	Fair	1 (0.3)	2 (0.6)
	Poor	3 (0.8)	1 (0.3)
**Client reported following conditions at visit, n (%)**			
	Diarrhea	2 (0.5)	0
	Coughing^a^	2 (0.5)	6 (2.0)
	Tuberculosis positive^c^	47 (22.6)	27 (6.5)

a Self reported adherence obtained from medical records defined as follows: good (<5% of doses missed); fair (5–15% of doses missed); poor (>15% of doses missed).

b Significant difference observed between intervention and comparison groups at p<0.05 using Chi-square test.

c Tuberculosis diagnosis and staging differed between the health care facilities; for this reason no further analysis of tuberculosis was conducted.

The main source of drinking water was either public or private piped water for 98% of intervention group participants and 88% of comparison group participants. All households stored drinking water in the home. At baseline, 48% of clients reported that they did not treat their drinking water. At baseline, before receiving the BCP, 29% of intervention group clients reported currently treating their drinking water with Wuha Agar compared to 13% of comparison group clients (p<0.05); 20% of stored water samples in the intervention group and 8% in the comparison group had detectable free chlorine residuals (p<0.05); baseline differences were likely a result of intermittent distributions of products in the community by non-governmental organizations.

At baseline, similar percentages of clients in the intervention and comparison groups reported having soap in the home (76% vs. 78%). A significantly higher percentage of clients in the intervention than in the comparison group reported presence of PuR (21% vs. 0%, p<0.05), condoms (24% vs. 12%, p<0.05), and albendazole tablets (16% vs. 2%, p<0.05) in the home, while significantly fewer intervention group clients than comparison group clients reported having Wuha Agar in the home (24% vs. 42%, p<0.05).

### Biweekly Home Visits

Over seven rounds of home visits (16 weeks), a significantly higher percentage of intervention group clients than comparison clients were observed to have Wuha Agar (91% vs 19%, p<0.001), PuR (73% vs 0%, p<0.001), condoms (60% vs 3%, p<0.001), soap (51% vs 36%, p<0.001), albendazole (69% vs 0%, p>0.001), and a BCP water storage container (65% vs 0%, p<0.001) in the home at all visits (Table 3). A similar percentage of intervention and comparison group clients had cotrimoxazole tablets in the home at all visits (53% vs 57%).

**Table 3:** Percentage of clients observed to have interventions in the home; reported and observed water treatment behaviors; and reported receipt of interventions, by intervention and comparison group, over 16 weeks of basic care package evaluation, Gonder and Debre Markos, Ethiopia, October 2009–January 2010.

Characteristic		Intervention Group(n = 376)	ComparisonGroup (n = 308)	p-value
**Interventions observed in home at every visit**				
Wuha Agar	341 (90.7)		59 (19.2)	<0.001^a^
PuR	276 (73.4)		0 (0.0)	<0.001^a^
Condoms	224 (59.6)		10 (3.3)	<0.001^a^
Soap	192 (51.0)		111 (36.0)	<0.001^a^
Cotrimoxazole	198 (52.7)		174 (56.5)	0.35
Albendazole	258 (68.6)		0 (0.0)	<0.001^a^
BCP container	243 (64.6)		0 (0.0)	<0.001^a^
**Water treatment**				
Reported treating water at every visit	177 (47.1)		5 (1.6)	<0.00^a^
Reported treating water with Wuha Agar at every visit	170 (45.2)		5 (1.6)	<0.001^a^
Water tested positive for free chlorine residual at every visit	152 (40.4)		3 (1.0)	<0.001^a^
Reported buying water treatment products at ≥5% of visits	5 (1.4)		2 (5.0)	0.15
Received free Wuha Agar at ≥5% of visits	19 (5.1)		51 (16.6)	<0.001^a^
**Reports of interventions purchased or received**				
Bought soap at ≥50% of visits	316 (84.0)		267 (86.7)	0.38
Bought soap at every visit	149 (39.6)		117 (38.0)	0.69
Received free soap at ≥10% of visits	26 (6.9)		35 (11.4)	0.04
Bought cotrimoxazole at ≥10% of visits	13 (3.5)		55 (17.9)	<0.001^a^
Received free cotrimoxazole at ≥50% of visits	6 (1.6)		39 (12.7)	<0.001^a^
Received free condoms at ≥15% of visits	3 (0.8)		16 (5.2)	<0.001^a^
Bought condoms at ≥10% of visits	20 (5.3)		12 (3.9)	0.47

a Remains significant at <0.001 after controlling for False Discover Rate [38].

**Figure 1:**
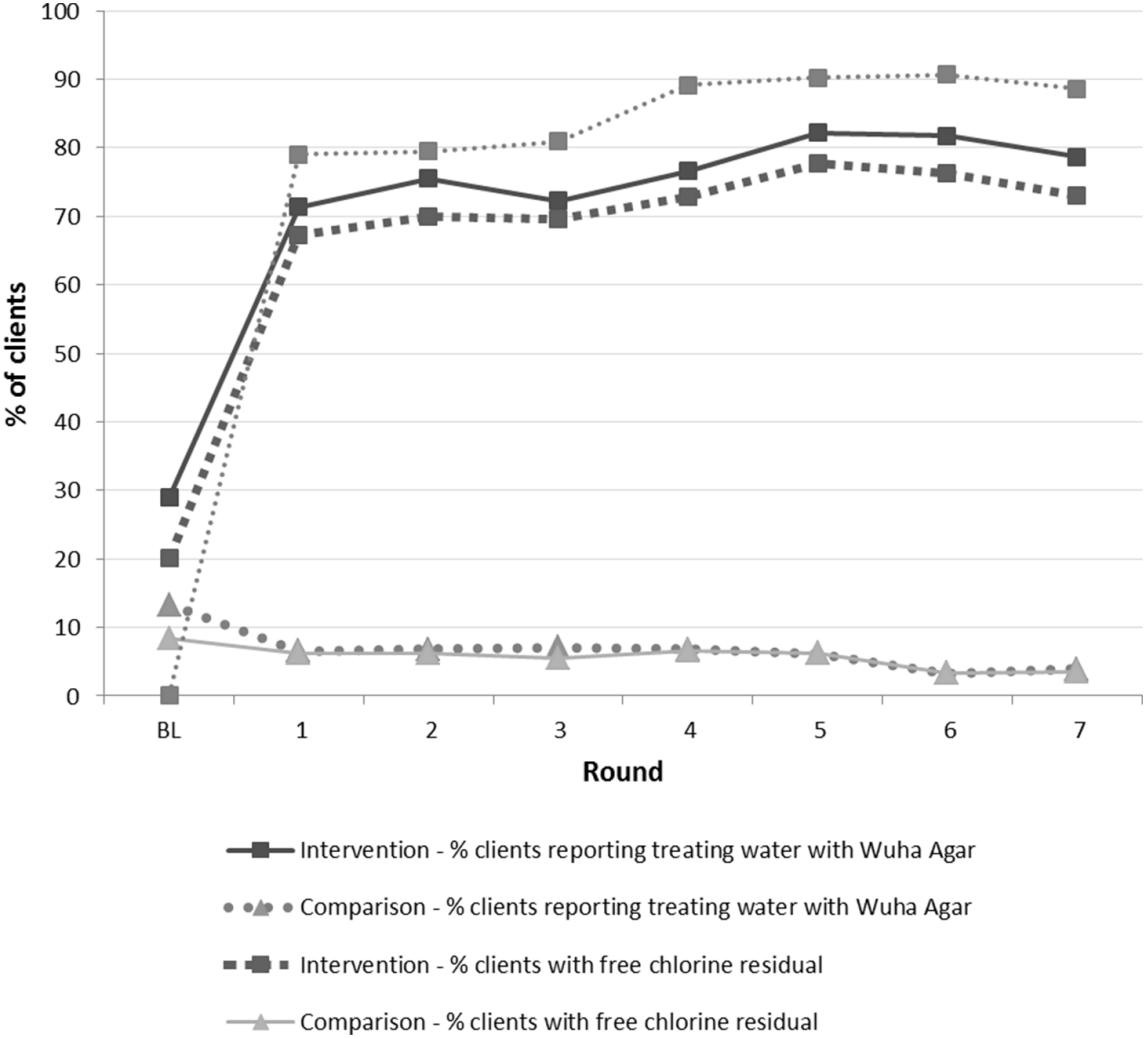
Percent of clients with reported water treatment with Wuha Agar, free chlorine residual in stored water, and observed BCP container, at baseline (before receiving the BCP) and by home visit round, by intervention and comparison group, basic care package evaluation, Gonder and Debre Markos, Ethiopia, October–January, 2010.

During home visits, the percent of intervention group clients reporting water treatment with Wuha Agar ranged from 71% to 82% and the percentage with free chlorine residuals detected in drinking water ranged from 67% to 78% (Figure 1). In the comparison group, neither reported Wuha Agar use, nor observed free chlorine residuals exceeded 10% at any home visit. Observed PuR in the home among the intervention group ranged from 83% to 90% during the home visits, with a median count of 24 sachets (range 3, 37) at all 7 home visits. Over 7 rounds of home visits, a significantly higher percentage of intervention group clients than comparison clients reported treating their water with Wuha Agar (45% vs 2%, p<0.001) and were observed to have free residual chlorine in stored water at all visits (40% vs 1%, p<0.001) (Table 3).

A similar percentage of intervention and comparison group clients reported during 50% or more of home visits that they had purchased soap in the preceding 2 weeks (Table 3). More comparison than intervention clients reported during at least 50% of home visits that they had purchased cotrimoxazole (18% vs 4%, p<0.001) or received free cotrimoxazole (13% vs 2%, p<0.001) in the preceding 2 weeks.

During the study, 75% of intervention group clients reported they were concerned that their friends or neighbors might learn they had received the BCP, which could signal their HIV status. However, only 8% reported feeling discriminated against because of the BCP, and 67% told friends or family about components of the BCP that they had received.

Intervention group clients were less likely than comparison clients to report illness from any cause (44% vs 67%, p<0.05) or at least one episode of febrile illness (28% vs. 38%, p<0.05) in the 48 hours preceding home visits (Table 4). Over the 14 days preceding home visits, intervention clients were less likely than comparison clients to report health facility visits for any illness (74% vs. 95%, p<0.05), respiratory illness (5% vs. 10%, p<0.05), or fever (9% vs. 15%, p<0.05). Intervention group clients reported fewer hospitalizations for any cause (1% vs. 3%, p = 0.12) and health facility visits for diarrhea (4% vs. 7%, p = 0.11) than comparison clients, but the differences were not statistically significant. The median illness score was 1.0 (range 0, 22) for intervention group clients and 3.0 (range 0, 33) for comparison group clients (p<0.05, Table 5).

**Table 4:** Percent of clients self-reporting one or more episodes of illness, health facility visit, and hospitalization by intervention and comparison group, over 16 weeks of basic care package evaluation, Gonder and Debre Markos, Ethiopia, October 2009–January 2010.

Characteristic^a^		InterventionGroup (n = 376)	ComparisonGroup (n = 308)	p-value
**Illness in previous 48 hours**	165 (43.9)		206 (66.9)	<0.001^c^
Diarrhea in previous 48 hours	37 (9.8)		39 (12.7)	0.27
Respiratory illness in previous 48 hours	53 (14.1)		50 (16.2)	0.55
Fever in previous 48 hours	105 (27.9)		117 (38.0)	<0.005^c^
**Health facility visits in previous 14 days**	277 (73.7)		292 (94.8)	<0.001^c^
Health facility visit for diarrhea in previous 14 days	14 (3.7)		20 (6.5)	0.11
Health facility visit for respiratory illness in previous 14 days	20 (5.3)		32 (10.4)	0.01^c^
Health facility visit for malaria in previous 14 days	5 (1.3)		3 (0.1)	0.74
Health facility visit for fever in previous 14 days	35 (9.3)		45 (14.6)	0.04
Health facility visit for STI in previous 14 days	3 (0.8)		1 (0.3)	–
Health facility visit for HIV care in previous 14 days^b^	30 (8.0)		67 (21.8)	<0.001^c^
Health facility visit for other illness in previous 14 days	93 (24.7)		111 (36.0)	0.001^c^
**Hospitalization in previous 14 days**	5 (1.3)		10 (3.3)	0.12
Hospitalization for diarrhea in previous 14 days	0 (0)		2 (0.7)	0.2
Hospitalization for respiratory illness in previous 14 days	0 (0)		1 (0.3)	0.45
Hospitalization for malaria in previous 14 days	0 (0)		0 (0)	–
Hospitalization for fever in previous 14 days	0 (0)		2 (0.7)	0.2
Hospitalization for STI in previous 14 days	0 (0)		0 (0)	–
Hospitalization for HIV care in previous 14 days^b^	0 (0)		2 (0.7)	0.2
Hospitalization for other illness in previous 14 days	5 (1.3)		10 (3.3)	0.12

a Illnesses spanning multiple home visits were counted each time they were reported over 16 weeks of basic care package evaluation.

b Health facility visits and hospitalizations for HIV care, including changes in ART medications, CD4 counts, etc. do not contribute towards the illness score.

c Remains significant at <0.05 after controlling for False Discover Rate [38].

**Table 5:** Total number of illnesses, health facility visits, and hospitalizations reported over 16 weeks of basic care package evaluation, and illness scores derived from these events, by intervention and comparison group, Gonder and Debre Markos, Ethiopia, October 2009–January 2010.

Characteristic	Intervention Group(n = 2,632)	ComparisonGroup (n = 2,156)	Weighting factor	Illness Score for Intervention Group	Illness Score for Comparison Group
**Illness in previous 48 hours**	653	946	1	653	946
**Health facility visits in** **previous 14 days**	220	310	2	440	620
**Hospitalization in previous** **14 days**	5	15	2	10	30
**Total illness score**				1,103	1,596
**Median illness score per** **patient (range)**				1.0 (0–22)	3.0 (0–33)

## Discussion

Findings of this study, which was the first to attempt to ascertain the health impact of a program to provide BCPs to PLHIV, suggested that recipients of the BCP were likely to have a lower illness score derived from self-reported illnesses, health facility visits, and hospitalizations than PLHIV who did not receive the BCP. Rates of reported diarrheal illness, and health facility visits or hospitalizations for diarrhea among intervention clients were also lower than in comparison clients, but disease rates were too low to provide the power to conclude that the differences were statistically significant. Nevertheless, the results are consistent with evidence from five high-quality studies, including four randomized controlled trials and one systematic review and meta-analysis which support the beneficial impact of interventions to improve household drinking water quality on the health of PLHIV and their families [5], [23]–[26].

The differences between the two groups in reported illness and health facility visit rates occurred following documentation of similar functional status, CD4 cell counts, duration on ART, adherence to ART, and adherence to cotrimoxazole prophylactic treatment among intervention and comparison clients at baseline, and in spite of the finding that a lower percentage of intervention clients than comparison clients were on cotrimoxazole prophylaxis at baseline. Furthermore, a higher percentage of clients in the comparison than the intervention group reported during home visits that they had received free Wuha Agar, soap, condoms, and cotrimoxazole from other health facility-based programs and had bought cotrimoxazole, all of which would likely diminish the differences in health outcomes between the two groups. Finally, these findings were observed despite relatively low rates of illness, which were likely a result of ART and cotrimoxazole prophylaxis [5], [6], and suggested that, even in a setting of low illness rates, the BCP may provide incremental clinical benefits to patients receiving these treatments. An alternative interpretation is that differences between the two communities may have accounted for the findings. Health findings were congruent with reported and confirmed use of BCP components documented in this study. Specifically, intervention clients were more likely than comparison clients to be observed with BCP components in their homes, including Wuha Agar, PuR, BCP water containers, condoms, soap, and albendazole. Similarly, clients who received the BCP were significantly more likely than comparison clients to report treating their stored drinking water with Wuha Agar and have free chlorine residuals in stored drinking water. By removing the economic barriers to access to these products through free distribution, [27]–[29] the program created an opportunity for participants to use them, and participants took advantage of this opportunity, as has been observed in at least one other evaluation [17]. Without free distribution, it is likely that use of different BCP components would have been lower in this population because two-thirds earned no income.

Economic barriers to sustained use of the BCP components could diminish or eliminate health gains observed in this study because only one BCP per client is provided and clients may not be able or willing to replenish consumable commodities in the BCP such as Wuha Agar, condoms, and albendazole. Research in other settings has shown that repeated purchase of inexpensive commodities in low income populations often does not take place, [27]–[29] even following a free trial [27], [30], [31]. To our knowledge, only one other evaluation, which took place in Uganda, has examined use of BCP components among PLHIV, and this study was conducted before depletion of the original supply of intervention components [18]. An evaluation of use of BCP components over time in both the Ethiopian and Ugandan programs is warranted and could help guide programming. In addition, an assessment of the costs and benefits of a program of free resupply of BCP commodities would be useful for guiding policy, [32], [33] particularly in view of evidence that integration of free health interventions into health services can serve as an incentive to increase use of services [34].

Some of the components of the BCP distributed in Ethiopia may be unnecessary. For example, soap was observed in a similar percentage of homes in both evaluation groups throughout the evaluation, and similar percentages of clients reported purchasing soap, suggesting that the BCP did not likely change access to or use of soap. In contrast, despite the baseline observation that a higher percentage of intervention than comparison households had PuR in the home, possibly as a result of previous distribution efforts by local organizations, use of PuR in this evaluation appeared to be low, as suggested by the observation that the median number of PuR sachets in intervention client homes remained at 24, the total number provided in the BCP, throughout the 7 rounds of home visits. One possible explanation for this observation is that most intervention clients had access to piped water, which tended to be clear and not in need of flocculation, and access to Wuha Agar for in-home chlorination. By eliminating items from the BCP that are already present or infrequently used in most households, resources could be more efficiently directed toward alternative interventions.

The finding that about half of clients in both groups had cotrimoxazole in the home at every visit, and that relatively few patients reported buying or receiving it for free, was of some concern. Prophylactic treatment with cotrimoxazole has been shown to reduce morbidity and mortality from opportunistic infections among PLHIV [6], including those on ART and has been recommended for inclusion in BCPs [35]. High adherence to recommended prophylactic treatment has been achieved in other programs [6], [11] and assuring regular access and use of the drug should be a priority.

This study had several important limitations. First, BCPs were not randomly distributed because that would have caused potentially disruptive, inequitable treatment of clients, logistical complications for nursing staff, and ethical concerns. We therefore chose to compare health outcomes in the context of a phased BCP distribution to intervention and comparison sites that were chosen on the basis of similar population characteristics. The resultant quasi-experimental design with just two study sites was subject to potential confounding factors, such as differences between the two study sites in geography; climate; demographic and socio-economic status; household water management habits; and health care delivery practices. Thus, we cannot rule out that the findings observed could have been due to differences between the two communities. However, while health care delivery and water treatment practices differed at baseline between the two groups, the large magnitude of difference observed between the two populations during the study would have been highly unlikely without the relatively intensive intervention that took place. Second, because of logistical and financial considerations, we were limited to two sites in one region of Ethiopia, so the evaluation population was neither representative of Amhara state nor the country as a whole and, therefore, different results might be observed in other populations. Donors providing major investments into interventions such as those described here should budget adequately for an evaluation component to avoid similar situations. Third, because all clients enrolled in the study lived within a 10 km radius of the town, information about clients living in more remote settings was unavailable. Fourth, biweekly home visits may have influenced responses to questions and observable household practices, resulting in a Hawthorne effect [36], but this effect would likely have impacted both the intervention and comparison groups. Fifth, because the evaluation was designed to evaluate the BCP as a whole we had limited ability to tease out the effect of specific interventions in the package on specific diseases, and could not elucidate which interventions in the package drove the findings. Finally, because of resource limitations, active surveillance was limited to 14 weeks; therefore we were not able to follow clients until consumable BCP components were exhausted, limiting our ability to determine whether their use was sustained over time. In addition, the short duration of the study made it difficult to assess whether illness patterns varied seasonally and over time, or whether there was attrition in the health impact of water treatment interventions over time, which has been observed elsewhere [37].

Because of these limitations, the findings of this evaluation must be interpreted with caution. However, results of this study suggest that, taken as a whole, the BCP is a promising method of increasing access to inexpensive interventions that can reduce the risk of disease and improve the quality of life of PLHIV. This evaluation also highlighted the importance of evaluating BCP programs in order to assure maximum health impact and optimal use of scarce resources.
